# *Naegleria*: a classic model for de novo basal body assembly

**DOI:** 10.1186/s13630-016-0032-6

**Published:** 2016-04-04

**Authors:** Lillian K. Fritz-Laylin, Chandler Fulton

**Affiliations:** Department of Cellular and Molecular Pharmacology, University of California, San Francisco, CA 94158 USA; Department of Biology, Brandeis University, Waltham, MA 02454 USA

## Abstract

The amoeboflagellate *Naegleria* was one of the first organisms in which de novo basal body/centriole assembly was documented. When in its flagellate form, this single-celled protist has two flagella that are templated by two basal bodies. Each of these basal bodies is structurally well conserved, with triplet microtubules and well-defined proximal cartwheel structures, similar to most other eukaryotic centrioles. The basal bodies are anchored to the nucleus by a single, long striated rootlet. The *Naegleria* genome encodes many conserved basal body genes whose expression is induced prior to basal body assembly. Because of the rapid and synchronous differentiation from centriole-less amoebae to temporary flagellates with basal bodies, *Naegleria* offers one of the most promising systems to study de novo basal body assembly, as well as the mechanisms regulating the number of centrioles assembled per cell.

## The organism

**Fig. 1:**
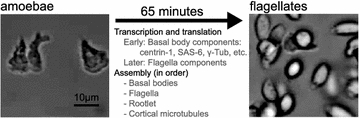
*Naegleria* differentiation. Amoebae can differentiate into flagellates, during which time they assemble basal bodies, flagella, flagellar rootlets, and a cortical microtubule cytoskeleton de novo. This process takes about an hour, and includes transcription and translation of basal body and* flagella* genes, including flagellar tubulin [5–9]. This process has been experimentally optimized to be highly synchronous and temporally reproducible [2, 3, 20, 25]

*Naegleria gruberi* is a free-living protist easily isolated from freshwater sources around the world [1–3]. *Naegleria*’s reproductive form is a 15-µm predatory amoeba that feeds on bacteria (Fig. 1). However, when faced with environmental signals such as nutritional, temperature, osmotic, and/or pH shifts, *Naegleria* undergoes an astounding metamorphosis from a crawling amoeba to a streamlined flagellate capable of swimming for several hours before reverting to an amoeba [2, 3]. Only the amoebae reproduce, and their mitosis involves no centrioles [4]. The amoeba-to-flagellate differentiation requires de novo assembly of basal bodies and flagella, including transcription and translation of their molecular components, even including tubulin (Fig. 1) [5–9]. Despite the complexity of this task, *Naegleria* cells accomplish the amoeba-to-flagellate conversion in about an hour [2, 3]. This developmental feat led to one of the first discoveries of de novo basal body assembly [4], at a time when even the concept of de novo centriole assembly was met with scepticism. To this day, one of the most interesting features of *Naegleria* centrioles is the speed at which differentiating cells turn on the genes, synthesize the proteins, and assemble two canonical basal bodies without any pre-existing “template” precursors. *Naegleria* synthesizes and assembles centriole components only during the transition to its temporary flagellate form; in the laboratory, at least, it can live for years as reproducing amoebae or resting cysts without ever using centrioles.

*Naegleria* has been developed as a model to study its incredibly rapid, synchronous, and reproducible differentiation from one cell phenotype to a very different one. Protocols have been developed for straightforward control of this process [2, 3], a methodology that opened the door to understanding the roles transcription and translation play in de novo centriole assembly [10], and tracing the expression, translation, and localization of individual proteins during differentiation [5–8]. More recently, genome sequencing has revealed that *Naegleria* has many canonical centriole/basal body genes, and microarray analysis of differentiation has also led to the prediction of novel centriole genes [9, 11].

*Naegleria* is a member of the heteroloboseans, a clade composed of a wide variety of amoebae, flagellates, and amoeboflagellates, of which *Naegleria* is the best-studied example [11]. The heteroloboseans are distantly related to two other groups, the jacobids, and the euglenozoans that include the parasitic trypanosomes [12]. The ancestor of these three clades diverged from other eukaryotic lineages somewhere during the past 1–3 billion years [11, 13].

Despite the eons that separate *Naegleria* from animal and fungal lineages, analysis of its fully sequenced genome indicates that *Naegleria* represents a sophisticated and surprisingly complex modern eukaryote, with about 16,000 genes including complete actin and microtubule cytoskeletons, mitotic and meiotic machinery, transcription factors [14], membrane trafficking, extensive networks of signaling machinery (including hundreds of protein kinases and small GTPases), and both aerobic and anaerobic metabolic pathways [11].

The genus *Naegleria* has about 40 species that are defined mainly by differences in extrachromosomal DNA sequences [15]. Many of these have very similar life histories, although there are some less-studied species that appear to have other options in their life cycles (such as division in flagellates [1]). Clonal strains of two morphologically very similar free-living species have been used for almost all studies of basal body development and form. One is *N. gruberi* strain NEG (the strain for which we have a draft genome [11]); the other was also known as *N. gruberi* strain NB-1 until a difference in ITS sequence caused it to be redefined as *N.pringsheimi* [15]. Herein when we refer to *Naegleria* we are referring to studies in strains NEG and NB-1. (The opportunistic human pathogen *N. fowleri* has a similar life cycle, and when it forms flagellates the basal bodies appear to be formed de novo [16, 17]).

## Basic basal body structure

**Fig. 2:**
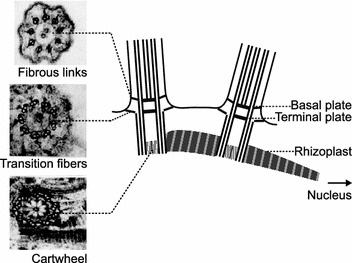
*Naegleria* basal body structure. Schematic of both *Naegleria* basal bodies drawn in longitudinal section, including the single rhizoplast (striated rootlet) that connects both basal bodies to the nucleus. Electron micrographs of cross sections of the flagellar-basal body apparatus highlighting Y-shaped links (*top*), transition fibers (*middle*) and cartwheel are adapted from figure 5 of [18]

Mature *Naegleria* flagellates typically have two basal bodies that are anchored at the plasma membrane and template motile flagella [18]. The two basal bodies appear structurally equivalent, with triplet microtubules and a clear luminal cartwheel at the proximal end (Fig. 2) [18]. Consistent with this canonical centriole ultrastructure, the *Naegleria* genome encodes many conserved centriole components, including γ-, δ-, and ε-tubulins, and SAS-6 [11]. These and other core components are readily recognized, although some *Naegleria* orthologs have extensively diverged from those of commonly studied species.

Based on a seminal electron microscopy study of *Naegleria* basal bodies and flagella [18], transition zones also appear well conserved. Although electron micrographs revealing details of the lumen of the transition zone are not available, the published data clearly show electron densities representing both basal and terminal plates [18]. Fibrous links between microtubule doublets and the membrane can be seen at the level of the basal plate, likely corresponding to the Y-shaped links seen at this location in other organisms, connecting microtubule doublets to the ciliary neck. Proximal to the terminal plate, fibers radiate from microtubule triplets into the cytoplasm, which are likely transition fibers [18].

## Additional basal body structures or accessory structures

*Naegleria*’s dual basal bodies are connected to its nucleus by a slender, long (up to 15 microns) striated rootlet called a rhizoplast (Fig. 2) [18–20]. One end of the rhizoplast is tightly adhered to the proximal end of the basal bodies via a striated wedge-shaped structure, while the other end runs along the nucleus, terminating in a pocket within the nuclear envelope [18].

The strength of the attachment of the rhizoplast to the basal bodies is evident by the ability of the two to be purified intact [19, 21]. Even the complex of nucleus and flagellar apparatus (basal bodies, rootlets, flagella) are sufficiently attached to be co-isolated [18]. Purified rhizoplasts appear to be at least 50 % composed of a single 170KD protein, and have been suggested to be related to striated ciliary rootlets of other organisms [19, 21]. The major rootlet protein is synthesized de novo during differentiation, and the rootlet is assembled 5–6 min after the flagella become visible [20].

## Basal body origins

In *Naegleria,* basal bodies are transient structures, assembled during the amoeba-to-flagellate differentiation, functional for several minutes to hours, and then disassembled during the de-differentiation to the amoeboid form [2, 3, 8]. Electron microscopy studies of synchronously differentiating cells indicate that both basal bodies are built within minutes, about 10 min before flagella emerge [4]. This rapid de novo basal body assembly has been of interest for some time, and there are a number of studies focused on understanding the required molecular events.

Studies of *Naegleria* orthologs of known basal body proteins (Northern blots to measure their mRNAs, and Western blots and immunoflorescence using affinity-purified polyclonal antibodies raised to *Naegleria* proteins) along with other experiments, including chemical inhibition of translation, have shown that *Naegleria* basal body assembly occurs by stepwise assembly of conserved components that are transcribed and translated de novo [3, 4, 6–9, 22]. In several cases, it is clear that a cluster of basal body genes are coexpressed earlier in differentiation than the cluster of genes required for flagellar assembly, events defined in detail for centrin-1, SAS-6, and γ-tubulin [7, 8]. Together, these studies indicate that *Naegleria* basal body assembly proceeds in roughly the same order of events as during centriole assembly in *Chlamydomonas* or human cells. This conclusion is supported by full genome transcriptional profiling showing robust and rapid induction of known centriole genes during differentiation [9].

## Basal body life cycle and other functions

*Naegleria* basal bodies, like the rest of its cytoplasmic microtubule cytoskeleton, are assembled during the transition to the flagellate form and disassembled upon transition back to an amoeba [2, 3, 20]. *Naegleria* undergoes mitosis and cytokinesis as an amoeba, where there are no centrioles or basal bodies present [4, 23, 24]. It therefore represents an interesting case of centriole assembly outside of the cell cycle. Because *Naegleria* routinely reproduces for hundreds of generations in its amoeboid form without ever building or containing a centriole/basal body [4, 25], this organism clearly does not require a basal body or centriole for its normal growth. Mitosis in *Naegleria* is intranuclear, and the microtubules do not focus to the poles [4, 24, 26]. It is clear that the basal body does not assume the role of a centrosome, and there is no hint that any other structure serves to focus the mitotic microtubules.

However, in addition to templating the flagella [18], the basal bodies do seem to act as microtubule organizing centers in the flagellate, where a focus of γ-tubulin enrichment has been observed, from which emanates a large “cage” of microtubules which follows the cortex of the cell [8, 27, 28].

A genus of free-living amoeboflagellates closely related to *Naegleria*, *Tetramitus*, shows some striking differences from *Naegleria*. Like *Naegleria*, *Tetramitus* can differentiate from centriole-less amoebae to flagellates, in this case with four basal bodies and four flagella [2, 29]. The differentiation is slower, and requires a microaerobic environment [30]. Most strikingly, the flagellates can also become stable and reproduce, so that this species can assume two stable, reproducing phenotypes: amoebae and flagellates [2]. The ultrastructure of the flagellates has been described, and preliminary observations suggest that division in the flagellates is also acentriolar, and in particular that the basal bodies do not appear to participate in division [31, 32]. Surprisingly, this fascinating genus has been little studied to date.

## Identification of basal body components

Centrin has long been known to be associated with *Naegleria* basal bodies [7, 33], which have been more recently shown to contain SAS-6 [8]. Although proteomics of purified *Naegleria* basal bodies has not been reported, the mass induction of basal body genes during differentiation has been used to predict conserved and novel basal body proteins, including: δ- and ε-tubulins, SAS-4/CenP-J/CPAP and SAS-6, POC1, POC11, POC12, POC16, MKS1, and MKS3 [9]. *Naegleria*, like other eukaryotic species with motile flagella, also has conserved Pix proteins [34].

## Notable basal body findings

As has been discussed above, *Naegleria* was one of the first reported cases of de novo basal body assembly [4], and for decades remained the best-studied example. It was also by studying *Naegleria* differentiation, in particular the induction of α- and β-tubulin isoforms specific to flagellates, that led to the origin of the multitubulin hypothesis, which predicted the existence of multiple types of tubulin that would be used to build different cellular structures [5]. Both flagellar α- and β-tubulins, which are incorporated into basal bodies, flagella, and cortical microtubules, undergo highly regulated synthesis during differentiation [3, 5, 22, 35, 36]. Evidence has been presented that another, very divergent, α-tubulin is used for mitosis in *Naegleria* [37].

An area of great promise for future research in *Naegleria* is how the majority of differentiating *Naegleria* cells assemble exactly two basal bodies and two flagella. There are already some provocative observations in the literature that hint at an interesting counting mechanism.

*Naegleria* strain NEG is normally diploid (2n) [11], but in culture it often becomes tetraploid (4n), presumably due to failure of mitotic nuclei to separate [2] (p. 459). While the diploid strains tend to have two flagella (2n-2f), the tetraploids initially tend to have four flagella (4n-4f). This configuration is metastable, however, and after some growth in culture tetraploid cells tend to revert to forming two flagella upon differentiation (i.e., 4n-2f). In this state, they look very similar to strain NB-1, which is a stable tetraploid that typically makes two flagella (i.e., 4n-2f). In both cases, 4n-2f cells seem to have looser control over their counting, with around 20 % flagellates having 3–4 flagella, compared to only 2 % of 2n-2f NEG flagellates [2] (p. 413). These simple observations are easily reproduced [2, 25], but perhaps more challenging to understand. While ideas of possible precursors that divide along with cell division are appealing [38] (p. 199), they do not seem necessary since known proteins seem sufficient to nucleate the formation of a new basal body independent of any precursor structure (e.g., [39, 40]).

Strikingly, sublethal temperature shocks at appropriate times during differentiation can dramatically increase the number of basal bodies and flagella that *Naegleria* assembles [41, 42]. For example, on average strain NB-1 normally assembles 2.2 flagella. However, after a 38° temperature shock, this average rises to 4.5, with a range of up to 18 flagella on a single cell [41]. These multiflagellate cells display disorganized swimming and tumbling. When these flagellates revert to amoebae in the same nonnutrient environment, they immediately redifferentiate without division, but with only the normal number of flagella (average of 2.1) [41]. Why heat-shock temporarily alters flagellar number, as well as the nature of the normal control mechanism, remain interesting challenges for future investigation.

In three published reports from JooHun Lee’s laboratory, it has been suggested that a novel entity regulates *Naegleria* basal body assembly in an unprecedented manner [43–45]. Their work presents evidence that *Naegleria* amoebae maintain a novel protein complex through numerous generations. This complex, containing a *Naegleria* transacetylase protein, is reported to accumulate γ-tubulin, pericentrin, and myosin II. The resulting “GPM” complex, present in amoebae, moves to the site of basal body assembly, and provides the focus where two basal bodies form de novo. Then the complex (including γ-tubulin) leaves the site of basal body assembly, travels to the other end of the cell, and disassembles, leaving the basal bodies behind. In this study, the presence of γ-tubulin is used to build the hypothesis that the complex might transiently nucleate the start of basal body assembly. Although provocative, the reliance on mammalian antibodies without properly defined epitopes in *Naegleria* to trace the movement and fate of the GPM complex leaves room for serious disagreement with these findings. In the experience of our laboratories, *Naegleria* proteins are sufficiently divergent from other species that the immunofluorescence signal when using heterologous antibodies (if there is any) is almost always to unknown antigens, or proteins trapped at the posterior end of amoebae (e.g., [8]). Specifically, both our labs have tried heterologous antibodies to γ-tubulin, without success. This is in stark contrast to results obtained by using affinity-purified antibodies raised to the single *Naegleria* γ-tubulin gene product. These antibodies reveal that γ-tubulin is localized to the basal bodies during their assembly, and remains stably localized there—parallel to the result observed for γ-tubulin in other species [8]. In addition, our results indicate that γ-tubulin, like other basal body proteins, is not present in amoebae: the mRNA for γ-tubulin is induced early in differentiation [9], and γ-tubulin antigen accumulates as the basal bodies are assembled [8]. The fact that Lee’s results show the heterologous antibody epitopes are already present in amoebae, and go on to dissociate from the basal bodies, make it seems likely to us that the recognized epitope is not γ-tubulin. In their most recent paper [44], Lee et al. used a new antibody to a *Naegleria* γ-tubulin peptide, but in immunogold electron microscopy found that this antibody did not colocalize with the structure recognized by the heterologous γ-tubulin antibody they had used to define the GPM complex. (Similar objections apply to the heterologous pericentrin antibody they used; in this case it is also unknown what epitope is staining, and no pericentrin gene has been curated in the *Naegleria* genome). While the Lee laboratory’s ideas are provocative and interesting, resolving the issues caused by heterologous antibodies as well as more precise colocalization studies are essential to understanding their results. We hope these issues can be resolved in the near future.

Given the current interest in control of centriole formation, we would love to be able to discuss the role of individual genes in the control of *Naegleria* basal body assembly. For example, in animal cells there have been a series of key papers dissecting the role of polo-like kinase 4 (PLK4) in the control of centriole assembly and number (e.g., [46, 47]). In these animal cells, PLK4 localizes to existing centrioles and there becomes activated and appears to regulate the normal assembly of a single new centriole. In addition, overexpression of PLK4 can induce de novo centriole formation. One can imagine such roles for PLK4 in the rapid formation of basal bodies during *Naegleria* differentiation, but so far no *Plk4* gene has been recognized in the *Naegleria* genome. This could be due to genetic divergence, but a comparative study indicates that orthologs of *Plk4* may be limited to Ophisthokonts (animals and fungi) [48]. While *NaegleriaPlk1* might play the role of *Plk4* in the amoeboflagellate, any role of polo-like kinases in this system remains a challenge for future research, particularly given the current lack of tools for gene manipulation in *Naegleria* cells.

## Strengths and future of basal body research in *Naegleria*

The ease of cell culture and incredible synchrony of differentiation give *Naegleria* great promise as a system to understand basic mechanisms of basal body assembly. However, the lack of tools for molecular genetic analysis in *Naegleria* remains a very real hindrance. Despite efforts [49], there have not been any widely adopted methods of manipulating gene expression in this organism. However, the *Naegleria* genome encodes all the necessary molecular machinery for both meiotic recombination and RNAi, hinting that both forward and reverse genetic analysis should be feasible [11]. The recent publication of the *Naegleria* genome sequence [11], as well as full genome transcriptional profiling [9], opens the door to a new era of discovery and has led to a renewed interest and wider adoption of this classic model for basal body biology.

It is clear that the rapid de novo assembly of basal bodies, and the counting system that ensures that most cells assemble two basal bodies, makes *Naegleria* a unique system to study basal body assembly. The formation and reproduction of basal bodies in the two stable phenotypes of *Tetramitus* are also worthy of further study. All that is needed is that researchers meet the challenge of learning to apply molecular genetics to this fascinating system.

## Abbreviations

GPM complex: a complex containing gamma-tubulin, pericentrin, and myosin II
MKS: Meckel-Gruber syndrome
PLK: polo-like kinase
POC: proteome of centriole
SAS: spindle assembly abnormal
